# The evaluation of functional results before and after laparoscopic Heller myotomy for achalasia: a single center experience 

**Published:** 2017

**Authors:** Abdollah Pooshani, Mojgan Frootan, Saeed Abdi, Somayeh Jahani-Sherafat, Freshteh Kamani

**Affiliations:** 1 *Gastroenterology and Liver Diseases Research Center, Research Institute for Gastroenterology and Liver Diseases, Shahid Beheshti University of Medical Sciences, Tehran, Iran.*; 2 *Basic and Molecular Epidemiology of Gastrointestinal Disorders Research Center, Research Institute for Gastroenterology and Liver Diseases, Shahid Beheshti University of Medical Sciences, Tehran, Iran*; 3 *Basic and Molecular Epidemiology of Gastrointestinal Disorders Research Center, Research Institute for Gastroenterology and Liver Diseases, Shahid Beheshti University of Medical Sciences, Tehran, Iran*; 4 *Taleghani hospital, Shahid Beheshti University of Medical Sciences, Tehran, Iran*

**Keywords:** Achalasia, Heller myotomy surgery, Free Drinking pressure, dysphasia, Iran

## Abstract

**Aim:**

The aim of this study was to evaluate and compare the functional results before and after laparoscopic Heller myotomy for Iranian patients with achalasia.

**Background:**

Achalasia is a severe neuromuscular disorder of the esophagus, characterized by the loss of peristalsis and an inability of the lower esophageal sphincter (LES) to reach optimal relaxation.

**Methods:**

In this cross sectional study, patients who underwent Heller myotomy for achalasia via laparoscopy in Taleghani Hospital Tehran, Iran were evaluated. Symptoms including pressure of residual, integrated relaxation sphincter (IRP), pressure of free drinking, pressure of LES, dysphasia score and peristalsis movement was measured and recorded by manometry before and after (2 months) treating with Heller myotomy.

**Results:**

In this study, 23 patients with achalasia (12 females and 11 males) with a mean age of 30±3.5 years (minimum 20, maximum 44 years) who met the inclusion criteria of the study were examined. Results of this study showed that, all the diagnostic criteria that were measured before the treatment was significantly different from after the treatment (P<0.05). The average decline in LES, IRP, Residual Pressure, Free drinking esophagus, and dysphasia score were 23.1 mmHg, 16.24 mmHg, 18,7 mmHg, 18.9 mmHg, and 5.0 unit, respectively. Also the average increase of the peristalsis movement was 8.26±13.7 in 8 patients.

**Conclusion:**

Considering the results of Heller myotomy surgery can be as a treatment of choice for achalasia. Free Drinking pressure can be a suitable criteria after treatment for evaluation and prediction of the reducing the dysphasia score after the surgery.

## Introduction

 Achalasia is a rare esophageal motility disorder, which is characterized by failure of the lower esophageal sphincter (LES) to relax and to some extent by a lack of peristalsis in the tubular esophagus (1). The exact pathophysiology of achalasia has not been fully understood, but functional loss of ganglion cells in the myenteric plexus. In histological evaluation of damaged esophagus, inflammation in myenteric network with the loss of cells and fibers of myenteric network is evident (2).

 Achalasia typically occurs in adults aged 25 to 60 years old. It is extremely rare in children (5%), and occurs equally in both genders with prevalence that ranges up to 1 per 10,000 persons (3, 4). The most common symptoms of achalasia in more than 80% of patients are usually difficulty with swallowing, dysphagia, regurgitation, chest pain and weight loss (5, 6). These symptoms are due to the absence of peristalsis in esophageal, failure relaxation LES and high pressure in the lower esophageal sphincter (LES) on swallowing (7, 8). The diagnosis of achalasia is made by using evidence from endoscopy, barium swallow (esophagram), and esophageal manometry indicating an absence of peristalsis, high pressure in the LES, and failure of the LES to relax on swallowing (9). 

Although the etiology of achalasia is still unknown, the mainstay of therapy is focuses on the LES degradation to decrease sphincter pressure to improve esophageal emptying by gravity (10). All treatment for achalasia is palliative. LES degradation can be achieved by pneumatic dilatation (PD), Laparoscopic Heller myotomy (LHM), botulinum toxin injection, pharmacotherapy and peroral endoscopic myotomy (POEM) (11). Pharmacotherapy is usually unsuccessful. Also, PD with balloons is usually less successful in young patients (12). The balloon dilation treatment (15-58%) effective at 10 years and botox injection (85% initially effective, but only 30% at one year) are not effective than LHM (80-100% effective at 5 years, and 80% at 15 years with only 0.1% risk of mortality) that has been demonstrated by several studies (13). LHM has been developed as a non-incision, minimally invasive endoscopic treatment becoming the most effective option for achalasia (14). LHM recovery is more rapid and length of hospital stay reduced after minimally invasive cardio myotomy compared with conventional open surgery. LHM offers the potential benefit of reduction in the need for multiple procedures and may provide better overall symptom resolution with 80% long-term response (15, 16).

 There is no more clinical record regarding the functional results for achalasia in the Iranian population. Therefore, the purpose of this study was to evaluate and compare the functional results before and after (2 months follow-up) laparoscopic Heller myotomy for the treatment of achalasia in a referral center in Tehran, Iran. 

## Methods

**Study design**

After institutional review board approval was obtained, all patients with a diagnosis of achalasia who underwent laparoscopic Heller mytomy at Gastrointestinal (GI) ward of Taleghani Hospital (referral center of gastrointestinal disorders in Tehran, Iran) were reviewed. Patients with ages under 50 years were eligible for enrollment in the study with a diagnosis of achalasia that underwent laparoscopic Heller mytomy without any peristalsis. The diagnosis of achalasia was made by manometry and on the basis of the absence of peristalsis in esophageal, failure relaxation of the LES and high pressure in the LES on swallowing (as inclusion criteria). Patients who were diagnosed with achalasia associated with esophageal or gastric carcinoma, pregnant patients, patients older than 50 years and subject with previous therapy history were excluded from the study. 

 Finally 23 patients who met the inclusion criteria for the study were selected. Informed patient consent was obtained prior to the procedures. Collected patients were completed a standardized pre-treatment measurement consisting of esophageal manometry, clinical symptom evaluation and assigned to undergo Heller myotomy. Demographic characteristic such as age, gender, and clinical features including pressure of residual, integrated relaxation sphincter (IRP), pressure of free drinking, pressure of LES, dysphasia score and peristalsis movement were recorded for each patient. To determine improvement in symptoms, patients were evaluated two months after myotomy and underwent an additional manometry. Esophageal manometry was performed by a gastroenterologist before and two months after the treatment of Heller myotomy in all patients. The degree of improvements in symptoms was recorded after the treatment of Heller myotomy in patients. This method was performed by 4-cannula water perfusion system (Medtronic Co, Polygraph HR, USA). The pressure sensors of a polyvinyl catheter (4.5 mm diameter) were placed 5 cm apart. The catheter was passed through the nostrils down to the esophagus, under local anesthesia. Intra luminal pressures were recorded by graph for windows version 2.04 function testing software on a polygraph. The LES pressure was studied by station pull-through technique. Relaxation of the LES was carried out by asking the patient to drink a sip of water. The distance between the LES and sensors was 5 cm. Incomplete or loss of relaxation of LES and simultaneous contraction confirmed the diagnosis of achalasia.

**Statically analyses**

Patients’ demographic, clinical features and response to treatment were analyzed by SPSS software (version 20, Chicago, IL, USA). The paired t-test was used to compare the criteria before and after treatment. Two-sided p-values less than 0.05 were considered significant. 

## Results

In this cross-sectional study, 23 patients with achalasia 12 females (52.2%) and 11 males (47.8%) with a mean age of 30±3.5 years (minimum 20, maximum 44 years) who met the inclusion criteria of the study were examined. Esophageal manometries were performed in all patients before and after (2 months) the laparoscopic Heller myotomy surgery. Pressure of residual, integrated relaxation sphincter (IRP), pressure of free drinking, pressure of LES, dysphasia score and peristalsis movement was measured and recorded.

**Table1:** Pre-and postoperative results of functional state of the esophagus assessment

	Pre-Treatment Mean ± SD	Post-Treatment Mean ± SD	Deference between pre and post-treatment Mean ± SD	P-value
LES^*^ (mm Hg)	31.95±11.3	8.78±6.1	23.17±10.6	0.0001
IRP^#^ (mm Hg)	21.69±8.2	5.45±4.7	16.24±7.08	0.0001
Residual pressure (mm Hg)	24.52±10.9	5.78±4.8	18.74±9.7	0.0001
Free drinking esophagus (mm Hg)	29.67±16.3	10.73±13.7	18.93±14.7	0.0001
Dysphasia score (unit)	13.21±0.6	8.13±3.4	5.08±3.5	0.0001
Peristalsis Movement	-	8.26±13.7	8.26±13.7	0.0001

* LES: lower esophageal sphincter;

# IRP: Integrated relaxation pressure

**Table2:** Number of patients based on dysphasia score and free drinking esophagus

		Groups based on free drinking esophagus		Total
		≤5 n (%)	>5 n (%)	
Groups based on dysphasia score	<5 n (%)	0 (0)	8 (100)	8 (100)
	≥ 5 n (%)	15 (100)	0 (0)	15 (100)
Total		15 (100)	8 (100)	23 (100)

*P*-value < 0.001

Results of this study showed that, all the diagnostic criteria that were measured before the treatment was significantly different from after the treatment (P<0.05). The average decline in LES, IRP, Residual Pressure, Free drinking esophagus, and dysphasia score were 23.1 mmHg, 16.24 mmHg, 18,7 mmHg, 18.9 mmHg, and 5.0 unit, respectively. Also the average increase of the peristalsis movement was 8.26±13.7 in 8 patients (35.8%) (Table1).

Dysphasia score decreased equal or more than 5 unit (≥5) in 15 patients (65.2%) and the other patients had less than 5 unit (<5) decreased (35.8%) after Heller myotomy surgery. So based on unit decreased of dysphasia score, patients can be divided into two groups; less than 5 (<5) unit and more than 5 (≥5) unit. Moreover, according to decline of the free drinking pressure after Heller myotomy surgery patients also can be divided into two groups; less than 5 (<5) mm Hg and more than 5 (>5) mm Hg (Table 2).

Results of this study showed that, all the diagnostic criteria that were measured before the treatments were significantly different from those of after the treatment (*P*-value <0.001).

## Discussion

The aim of the present study was to evaluate and compare the functional results of laparoscopic Heller myotomy (LHM) via both an overall clinical score and dysphagia score. This comparative evaluation was obtain by evaluated the clinical symptoms such as; LES, IRP, residual pressure, free drinking esophagus, peristalsis movement and dysphagia score before and after the LHM. The goal of the treatment for achalasia is to improve quality of life and this is most affected by dysphagia. The approach to achalasia management has regained interest and is likely to be modified due to recent development of LHM (17, 18). This surgical procedure is thought to be associated with less postoperative pain, shorter hospital stay, and fewer parietal complications, and is now considered by some authors as the first intent treatment (19, 20). 

Results of this study showed that, functional results assessed on an overall clinical score and dysphasia score improved 2 months after the laparoscopic HM. According to the results of the present study, in all subjects decreased postoperative dysphagia scores were observed in the following form the mean score of dysphagia from 13.21±0.6 in pre-treatment was dropped to 8.13±3.4 in post-treatment. This means that dysphagia score decreased equal or more than 5 unit (≥5) in 15 patients (65.2%) and the other patients had less than 5 unit (<5) decreased (35.8%). While peristalsis movement only occurred in 8 (35.8%) patients and it didn’t occurred in other patients. Another diagnostic criteria such as; ELS, IRP, residual pressure, free drinking esophagus measured before the treatments were significantly different from after the treatment (*P*-value <0.001). 

Our findings confirmed the results of study by Abdi et al. in 2013-2014 that reported the Heller myotomy is highly effective in relieving dysphasia in patients with achalasia and all the diagnostic criteria measured after the treatment were significantly different and improved (4) and also similar to another study by Shiino et al. which have reported the persistent dysphagia and postoperative gastroesophageal reflux are the most cited reasons for surgical failure but dysphagia relief is maintained in 85– 100% of Heller myotomy patients (21). Lyass et al. have shown that Heller myotomy is highly effective in relieving dysphagia in patients with achalasia (22). Moreover, Krishnamohan et al. in retrospective review and follow-up of 500 patients who underwent laparoscopic myotomy in 2013 reported that LHM is a safe operation. The long-term outcome is extremely effective and approximately one-third of patients have no evidence of persistent symptoms at follow-up (23).

Jeansonne reported outcomes of LHM for achalasia in patients who underwent surgery more than 10 years prior. Most patients in this study reported satisfaction 10 years after the operation and dysphagia scores at 10 years were not different from those together at short-term follow-up. Their data suggest that the efficacy of LHMis sustained at 10-year follow-up (24).

In conclusion, LHM can be carried out safely used for treatment of the patients with achalasia, leading to a substantial, long lasting improvement in symptoms (25). The laparoscopic approach is also beneficial with respect to postoperative pain, mobilization and duration of hospitalization. The use of intraoperative manometry can be used for assessment of efficacy for patients with achalasia and myotomy to be assessed in real time, with significant benefits for patients’ symptoms in subsequent follow-up.

## Conflict of interests

The authors declare that they have no conflict of interest.
